# Distinct enteric nervous system pattern in obstructed defecation syndrome and slow-transit constipation: a controlled digital pathology study

**DOI:** 10.1007/s00384-026-05209-4

**Published:** 2026-08-03

**Authors:** Tillmann Bedau, Aryan Mirzaie-Kuzehgarani, Joshy Madukkakzhy, Luisa Halbe, Alexander Quaas, Claudia Rudroff

**Affiliations:** 1 Department of Pathology, Medical Faculty and University Hospital, University of Cologne, Cologne, Germany; 2 Department of Visceral Surgery and Functional Lower GI Surgery, Evangelisches Klinikum Koeln Weyertal, Cologne, Germany; 3 Department for Urology, Uro-Oncology, Specialized Urologic and Robot-Assisted Surgery, Medical Faculty and University Hospital, University of Cologne, Kerpener Str. 62, 50937 Cologne, Germany

**Keywords:** Constipation, Colectomy, Defecation, Enteric nervous system, Neurogenic bowel, Surgical pathology

## Abstract

**Purpose:**

Chronic constipation comprises heterogeneous clinical entities, including slow-transit constipation (STC) and functional defecation disorders such as obstructed defecation syndrome (ODS). Although neuroenteric mechanisms are implicated, reproducible histomorphological correlates remain incompletely defined, partly due to methodological heterogeneity and lack of standardized quantification. The study characterizes the myenteric plexus morphology and neuroimmune cell distribution across clinically defined subgroups of functional bowel disorders using a standardized digital pathology approach.

**Methods:**

Retrospective observational cohort study at a tertiary colorectal referral center. A total of 100 patients after bowel surgery were included: ODS (*n* = 17), STC (*n* = 24), combined ODS + STC (*n* = 28), and a control cohort without clinically evident bowel motility disorders (*n* = 31). Digital morphometric assessment of ganglionic density and morphology, neuronal content (immunohistochemistry against MAP2, HuC/D), and the distribution of CD3-positive T-lymphocytes within and around myenteric ganglia were performed.

**Results:**

Ganglionic density differed significantly between groups (*p* = 0.002), with increased density in ODS, whereas STC values were comparable to controls. Neuronal content per ganglion was preserved across all groups as demonstrated by MAP2 and HuC/D analyses. In contrast, the presence of CD3-positive lymphocytes was reduced in all constipation groups, most pronounced in STC, affecting both the intra- and periganglionic compartments.

**Conclusion:**

Functional bowel motility and defecation disorders appear to show distinct neuroarchitectural and neuroimmune patterns. Increased ganglionic density in ODS contrasts with preserved neuronal content and reduced presence of T-lymphocytes, particularly in STC. These findings represent associations rather than proven disease mechanisms, derived from comparison with a surgical rather than a healthy control cohort. Standardized digital morphometry nonetheless provides a reproducible framework for phenotypic characterization of chronic constipation and may support future studies linking enteric structure to function.

**Trial registration:**

Clinicaltrials.gov (NCT05016700; 09/12/2020).

**Supplementary Information:**

The online version contains supplementary material available at 10.1007/s00384-026-05209-4.

## Introduction

### Heterogeneity of chronic constipation

Chronic constipation affects 25–30% of the general population [1, 2] and represents a clinically and biologically heterogeneous syndrome rather than a single disease [3–6]. Contemporary classifications distinguish between disorders of the colonic transit, anorectal dysfunction, and combined phenotypes, including slow-transit constipation (STC) and functional defecation disorders such as obstructed defecation syndrome (ODS). Although these conditions often overlap, they are not interchangeable but represent distinct pathophysiologic entities [3, 4]. Indeed, up to 50% of patients with ODS also present with delayed colonic transit, which may represent coexistent colonic motor dysfunction or arise secondary to pelvic floor dysfunction (PFD) [7–9]. Women are disproportionately affected [10], and bowel dysfunction symptoms frequently occur in association with pelvic organ prolapse (POP) and after childbirth [11, 12]. These observations highlight the complexity of chronic constipation and the need for phenotypic differentiation.

### Histopathological findings and clinical correlation

Histopathological studies have described a range of alterations in chronic constipation, particularly in slow-transit constipation (STC), including changes in neurotransmitter expression, a reduction in interstitial cells of Cajal (ICC), and features of moderate hypoganglionosis with decreased ganglionic density and size [13–16]. In addition, degeneration of both the myenteric plexus and extrinsic pelvic parasympathetic innervation has been reported, supporting a potential neuropathic component [17]. More recent quantitative studies using three-dimensional imaging have confirmed reductions in neuronal and ganglionic density in STC, suggesting structural involvement of the enteric nervous system [18].

In our previous work, we identified CD8-positive T-cell–associated injury of myenteric ganglia, indicating a potential neuroimmune mechanism [19]; however, this pattern was observed only in a subset of patients and could not be generalized.

Additionally, clinical symptoms may occur in the absence of detectable histological alterations, while conversely, similar morphological findings may be present in individuals without corresponding symptoms. This inconsistent relationship between histopathology and clinical presentation remains a major unresolved issue.

### Lack of standardized quantification methods

A major limitation of the existing literature is the heterogeneity of the methods. Although the London Classification and related consensus statements emphasize standardized tissue processing and quantitative assessment of the enteric nervous system [20], many studies remain limited by small cohorts, variable staining protocols, inconsistent definitions of ganglionic abnormalities, and inadequate comparator groups. Differences in sampling location and analytical methods further reduce comparability. A further, largely unresolved methodological issue is the choice of comparator tissue: because full-thickness bowel wall cannot be ethically obtained from healthy individuals, published studies, including the present one, are necessarily restricted to archival tissue from patients undergoing bowel resection for other indications, so that disease-related alterations within the comparator cohort itself cannot be fully excluded.

### Aim of the study

The aim of this study was to systematically characterize myenteric plexus morphology and neuroimmune cell distribution in full-thickness bowel specimen from patients with obstructed defecation syndrome (ODS), slow-transit constipation (STC), or combined ODS + STC using a standardized digital pathology workflow. The results were compared with a surgical control cohort without clinically evident bowel motility disorder.

## Materials and methods

### Study design and patient selection

Between June 2021 and April 2024, a total of 69 patients diagnosed with ODS, STC, or a combination of both underwent surgical intervention after the failure of conservative treatment. Preoperative diagnostic workup included colonic transit time measurement, MRI defecography, contrast enema, and, if clinically indicated, computed tomography of the abdomen. Based on the predominant clinical presentation and diagnostic findings, patients were classified into three subgroups: STC, ODS, and combined STC + ODS.

Surgical procedures consisted of left-sided colorectal resection or subtotal colectomy combined with a suture rectopexy. In female patients with symptomatic POP, surgery was complemented by interdisciplinary sacropexy of the middle pelvic compartment as previously described [21, 22].

A surgical control cohort consisted of 31 patients undergoing colorectal resection for conditions not associated with clinically evident bowel motility disorders, including diverticular disease, endometriosis, or colorectal cancer.

Clinical characteristics, including age, sex, body mass index (BMI), and comorbidities as assessed by the American Society of Anesthesiologists (ASA) classification [6], were recorded for all patients.

All patients provided written informed consent for surgery, data collection, analysis, and publication. The study protocol was approved by the local ethics committee (Ethikkommission der Aerztekammer Nordrhein, Duesseldorf, Germany; Registration number 2018344).

### Histopathological analysis

A representative formalin-fixed paraffin-embedded (FFPE) tissue block from the distal anastomotic ring containing myenteric plexus structures was selected per case (*n* = 100). The block was selected by a board-certified gastrointestinal pathologist based on the presence of full-thickness bowel wall with a clearly identifiable myenteric plexus on the routine diagnostic hematoxylin and eosin (H&E) section, using a single, anatomically standardized sampling site (the distal resection/anastomotic margin) in every patient. Block selection was based on tissue quality and plexus visibility rather than a priori knowledge of quantitative outcome parameters; formal blinding of the selecting pathologist to the clinical diagnosis was not performed, which is acknowledged as a potential source of selection bias.

Sections were stained using Elastica-van Gieson (EvG) for morphological assessment, including better detection of collagen fibers, and with immunohistochemistry (IHC) for microtubule-associated protein 2 (MAP2) and T-lymphocytes (CD3, Quartett. Clone QR004 1:200 EDTA). In a subset of 20 cases (*n* = 5 per group), additional HuC/D IHC (clone 16A11, Invitrogen, catalog #A-21271, dilution 1:200) was performed for nuclear-based neuronal quantification. Whole-slide images were generated using a standardized digital scanning protocol. Image analysis was performed using QuPath software. Myenteric ganglia were manually annotated using the polygon tool, and a polyline was drawn in each slide to indicate the length of the section(s) spanning the annotated ganglia. Automated shape feature analysis (e.g., area, diameter, circularity) was conducted on EvG-stained slides. MAP2 expression was quantified by pixel-based analysis as the proportion of DAB-positive staining relative to total ganglion area. MAP2 was selected as the principal quantitative neuronal marker because it enabled pixel-based whole-slide digital quantification across the full cohort, whereas HuC/D required manual nuclear counting and was therefore restricted to an exploratory subset. CD3-positive intraganglionic and periganglionic T-lymphocytes were identified and quantified using automated cell detection in QuPath. To assess the neuroimmune environment, ganglion annotations were expanded by 25 µm to define a standardized periganglionic region, allowing separate quantification of intra- and periganglionic CD3-positive T-lymphocytes.

HuC/D-positive neurons were manually counted within annotated ganglia. Mean number of neurons per ganglion and linear neuronal density (neurons/mm) were calculated. Ganglion annotation was performed by one observer (TB); concordance was counterchecked by two further observers (AQ, AMK). A formal reproducibility statistic (e.g., kappa) was not calculated, which is acknowledged as a limitation.

### Workflow and quantitative analysis of the myenteric plexus

**Fig. 1:**
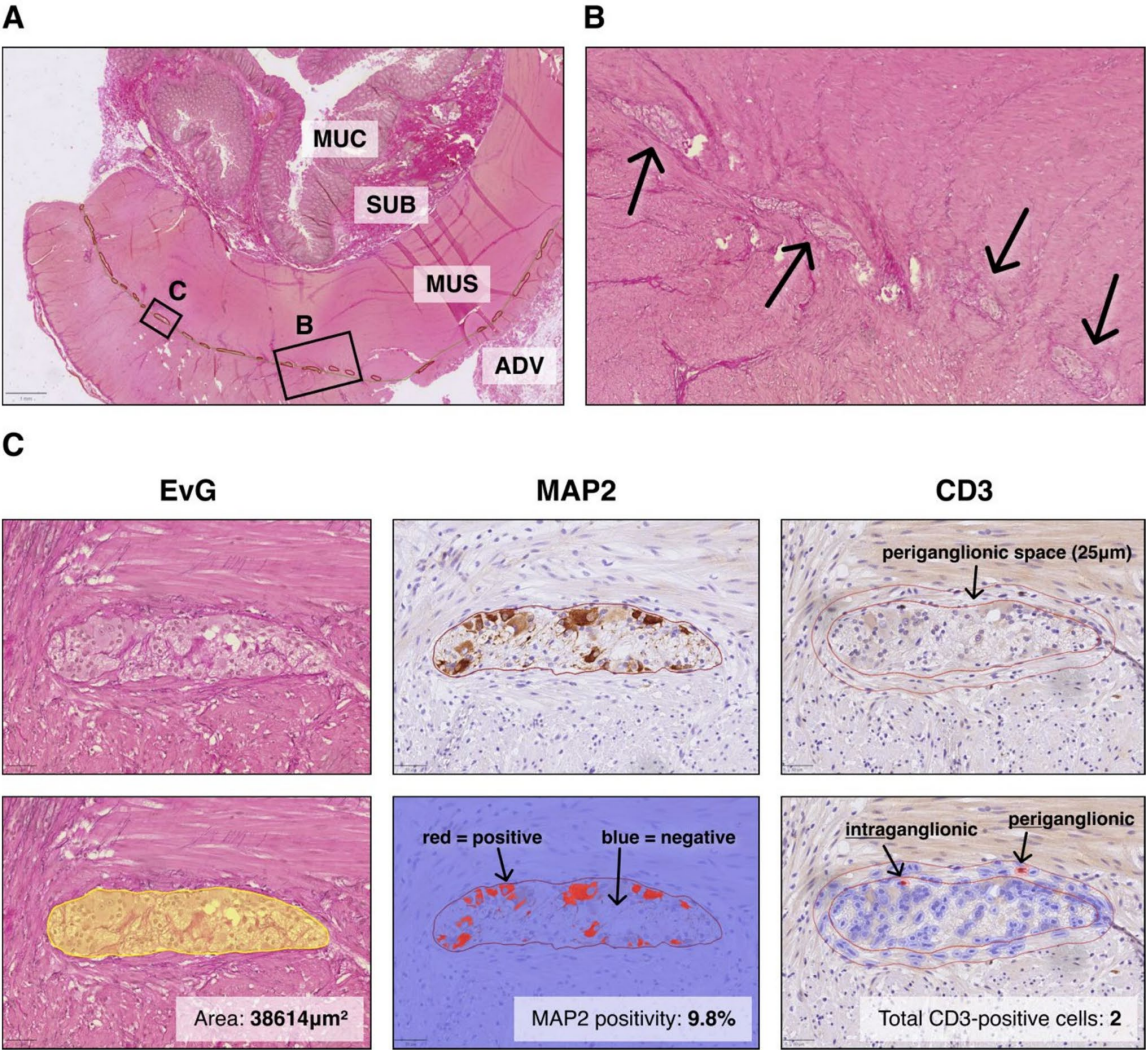
Histological analysis and digital quantification of the myenteric plexus. **A** Overview of a colon section stained with Elastica-van-Gieson (EvG), showing the four layers of the intestinal wall: mucosa (MUC), submucosa (SUB), muscularis propria (MUS), and adventitia (ADV). Representative areas of the myenteric plexus are indicated (B, C). **B** Higher magnification highlighting the myenteric plexus within the muscular layer; arrows indicate ganglionic structures. **C** Corresponding EvG, MAP2, and CD3 stainings of a representative ganglion with digital annotations. EvG delineates the ganglionic structure; MAP2 immunohistochemistry (IHC) highlights neuronal cells, and CD3 staining identifies T-lymphocytes within and surrounding the ganglion. A standardized periganglionic area expanded by 25 µm was defined to quantify intra- and periganglionic immune cell distribution

The workflow employed for histological analysis of the myenteric plexus is depicted in Fig. 1. For each included patient, a representative formalin-fixed, paraffin-embedded (FFPE) block was selected by a board-certified pathologist based on routine diagnostic hematoxylin and eosin (H&E) staining. Each selected block underwent EvG staining, as well as MAP2 and CD3 immunohistochemistry. These slides were subsequently digitized, and all ganglia within them were annotated digitally.

EvG staining, a connective tissue stain that delineates ganglia more clearly than H&E, was used to assess the overall architecture of the myenteric plexus. From these slides, we extracted data on ganglionic size and the total number of ganglia. This information was used to calculate ganglionic density, expressed as the number of ganglia per centimeter of annotated section, providing a relative measure of ganglionic presence. Linear ganglion density (ganglia/cm of annotated section) was chosen over an area-based estimate because the polyline annotation could be applied consistently along the plane of section on two-dimensional whole-slide images without requiring serial sectioning; a full stereological approach was not feasible within the retrospective the archival FFPE material for this study. Linear density may be influenced by factors unrelated to the true biological density of the enteric nervous system as will be discussed further in the “Limitations.” MAP2 staining, which specifically labels neuronal cells within ganglia, enabled an objective assessment of neuronal content. By digitally classifying DAB-positive pixels, we quantified MAP2 positivity for each ganglion as the proportion of DAB-positive pixels relative to the total ganglionic pixel count. Similarly, CD3 immunohistochemistry was used to measure T-lymphocyte infiltration within and around the ganglia. After annotating all ganglia, a periganglionic space—encompassing an area expanded by 25 µm from the original ganglionic boundaries—was defined. T-lymphocyte counts were obtained by quantifying DAB-positive (i.e., CD3-positive) cells in both the intra- and periganglionic spaces.

### Data analysis

Statistical analyses were performed using R (tidyverse package suite). Descriptive statistics were calculated for clinical variables. Adjustments for multiple analyses were applied where appropriate. Statistical analysis was performed using the Kruskal-Wallis test, followed by post hoc pairwise Wilcoxon tests with a Benjamini-Hochberg correction for multiple comparisons.

To complement *p*-values with a measure of the magnitude of group differences, Hedges’ *g* effect sizes with approximate 95% confidence intervals were calculated post hoc for the principal pairwise comparisons, using the group means, standard deviations, and sample sizes reported. As an exploratory, descriptive complement to the primary non-parametric analyses, results are reported in the “Results” (section “Exploratory effect size analysis”) and Supplementary Table 2.

To assess the internal homogeneity of the surgical control cohort, demographic characteristics and QuPath-derived histomorphological measurements were additionally compared across the three underlying control pathologies using the Kruskal-Wallis test for continuous variables and Fisher’s exact test for categorical variables, with Benjamini-Hochberg correction applied across the histomorphological measurements (Supplementary Tables 3 and 4).

Given the sample size and heterogeneity of the surgical cohorts, formal multivariable adjustment for potential confounders (age, sex, type of surgery, control pathology) was not undertaken (see “Limitations”).

## Results

### Patients’ characteristics

A total of 100 patients were included and categorized into four groups according to their clinical diagnosis: ODS (*n* = 17), STC (*n* = 24), combined ODS + STC (*n* = 28), and a control group (*n* = 31) undergoing bowel surgery for conditions unrelated to functional defecation disorders, including diverticular disease, endometriosis, and colon carcinoma. Detailed demographic, clinical, and resection-type characteristics are summarized in Table 1. Notably, subtotal colectomy was performed only for STC in four cases, whereas right hemicolectomy was performed exclusively in the control group (*n* = 6).

**Table 1:** Demographic and clinical characteristics of study population

Characteristic	Total (*N* = 100)	ODS (*N* = 17)	STC (*N* = 24)	ODS + STC (*N* = 28)	Controls (*N* = 31)
Sex—no. (%)					
Male	11 (11.0)	2 (11.8)	3 (12.5)	0 (0.0)	6 (19.4)
Female	89 (89.0)	15 (88.2)	21 (87.5)	28 (100)	25 (80.6)
Age					
Years—mean ± SD	57.1 ± 17.7	58.6 ± 18.1	59.6 ± 15.6	53.5 ± 14.6	57.6 ± 21.5
< 45 yr—no. (%)	26 (26.0)	4 (23.5)	6 (25.0)	6 (21.4)	10 (32.3)
45–65 yr—no. (%)	39 (39.0)	7 (41.2)	8 (33.3)	16 (57.1)	8 (25.8)
> 65 yr—no. (%)	35 (35.0)	6 (35.3)	10 (41.7)	6 (21.4)	13 (41.9)
BMI—mean ± SD	23.1 ± 3.3	23.0 ± 2.4	22.7 ± 2.4	22.8 ± 2.6	23.8 ± 4.7
ASA—no. (%)					
1	21 (21.0)	4 (23.5)	3 (12.5)	4 (14.3)	10 (32.3)
2	37 (37.0)	4 (23.5)	10 (41.7)	15 (53.6)	8 (25.8)
3	41 (41.0)	9 (52.9)	11 (45.8)	9 (32.1)	12 (38.7)
4	1 (1.0)	0 (0.0)	0 (0.0)	0 (0.0)	1 (3.2)
Type of resection—no. (%)					
(Sub-)total colectomy	4 (4.0)	0 (0.0)	3 (12.5)	1 (3.6)	0 (0.0)
Right hemicolectomy	6 (6.0)	0 (0.0)	0 (0.0)	0 (0.0)	6 (19.4)
Left-sided colorectal resection	90 (90.0)	17 (100.0)	21 (87.5)	27 (96.4)	25 (80.6)

Demographic and surgical data for 100 patients were categorized into four groups: obstructive defecation syndrome (ODS), slow-transit constipation (STC), combined ODS and STC (ODS + STC), and a control group for conditions without bowel motility disorders

*BMI* body mass index, *ASA* American Society of Anesthesiologists physical status classification, *SD* standard deviation

### Morphological characteristics of the myenteric plexus ganglia

**Fig. 2:**
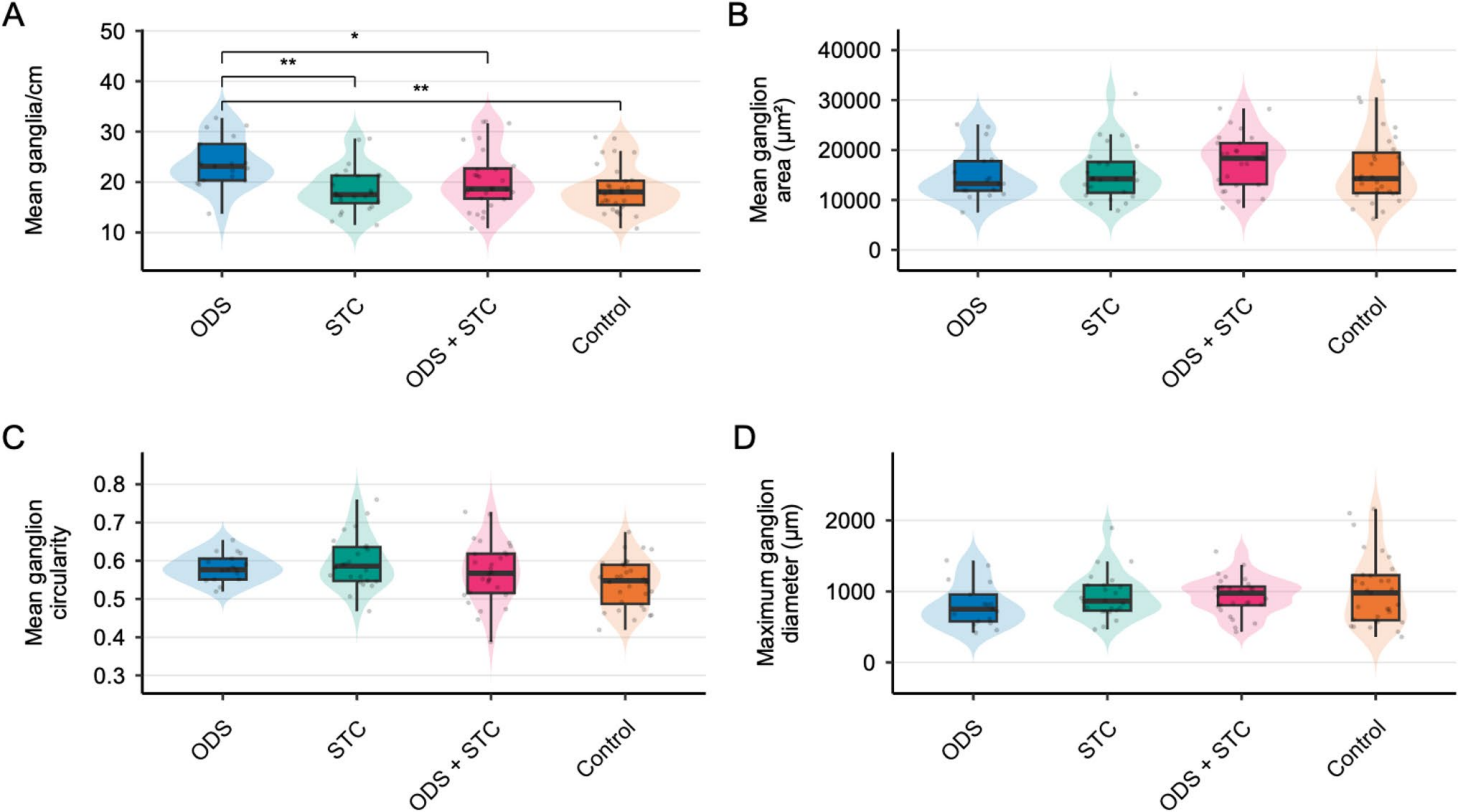
Enteric ganglion characteristics across patient groups. Comparison of enteric ganglion measurements in patients with obstructive defecation syndrome (ODS, *n* = 17), slow-transit constipation (STC, *n* = 24), combined ODS + STC (*n* = 28), and controls (*n* = 31). **A** Ganglionic density (ganglia/cm of annotated section length; see “Methods” for rationale and limitations of this normalization) was significantly higher in ODS compared with controls and STC (**p < 0.01). The ODS + STC group showed intermediate values, but significantly lower than ODS (*p < 0.05). **B**–**D** Mean ganglion area (µm^2^), circularity, and maximum ganglion diameter (µm) showed no significant differences. Data are presented as violin plots with superimposed boxplots (median, interquartile range) and individual data points

Ganglionic density differed significantly between the groups (*p* = 0.002; Fig. 2A). Patients with ODS showed a higher ganglionic density (23.8 ± 5.0 ganglia/cm) than control subjects (18.6 ± 4.7 ganglia/cm; *p* < 0.01). The combined ODS + STC group also demonstrated an increased density (20.5 ± 5.9 ganglia/cm; *p* < 0.05 vs. control), although values were lower than in the ODS group. In contrast, the STC group (18.5 ± 4.4 ganglia/cm) showed no difference compared with controls (18.6 ± 4.7 ganglia/cm).

Other morphological parameters were comparable across groups. Mean ganglionic area (Fig. 2B) did not differ significantly (*p* = 0.3), ranging from 15,015.2 ± 5138.2 µm^2^ (ODS) to 17,871.6 ± 5394.5 µm^2^ (ODS + STC). Ganglionic circularity (Fig. 2C) showed a borderline overall difference (*p* = 0.045), but no significant pairwise differences after correction for multiple testing; values were similar across groups (≈ 0.6 ± 0.1). Maximum ganglion diameter (Fig. 2D) also did not differ significantly, ranging from 813.1 ± 306.2 µm (ODS), 940.4 ± 264.8 µm (ODS + STC), 939.3 ± 323.7 µm (STC) to 1010.7 ± 492.7 µm (controls), respectively. Detailed values are provided in Supplementary Table 1.

Age-stratified analyses confirmed that differences in ganglion density were independent of age, whereas ganglion area and diameter were influenced by age rather than diagnostic subgroups (Supplementary Fig. 1). No significant effects of sex were observed for any morphological parameter (Supplementary Fig. 2).

### Neuronal content in the myenteric plexus

**Fig. 3:**
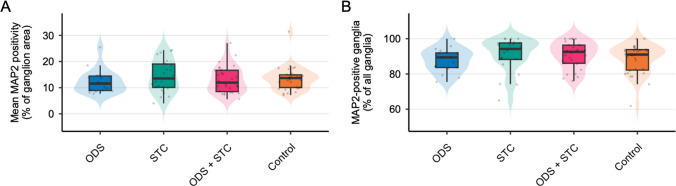
MAP2 positivity in enteric ganglia across patient groups. Comparison of MAP2 immunoreactivity in enteric ganglia between patients with obstructive defecation syndrome (ODS, *n* = 17), slow-transit constipation (STC, *n* = 24), combined ODS and STC (*n* = 28), and controls (*n* = 31). **A** Mean MAP2 positivity (percentage of ganglion areas) and **B** proportion of MAP2-positive ganglia did not differ significantly between groups. Data are presented as violin plots with superimposed boxplots (median, interquartile range) and individual data points

MAP2 immunoreactivity was analyzed to assess neuronal content within ganglia. Mean MAP2 positivity (Fig. 3A) was similar between groups (controls: 13.5 ± 4.6%; *p* = 0.6), ranging from 12.4 ± 4.6% in ODS to 14.4 ± 6.1% in STC patients. Similarly, the proportion of MAP2-positive ganglia (Fig. 3B) did not differ significantly (*p* = 0.2), ranging from 87.8 ± 6.8% (ODS) to 90.9 ± 9.5% (STC). These findings indicate that neuronal content within ganglia is preserved across all patient categories, despite differences in ganglionic density, particularly in ODS patients. Age- and sex-stratified analyses confirmed these results, with no significant effect on MAP2 immunoreactivity (Supplementary Figs. 3 and 4). Minor trends towards age- and sex-related differences in the percentage of MAP2-positive ganglia did not reach statistical significance (*p* = 0.0927 and *p* = 0.0837, respectively).

### Neuronal quantification by HuC/D immunohistochemistry

To complement the MAP2-based neuronal content assessment, HuC/D IHC was performed in a subset of 20 patients (*n* = 5 per group) to enable direct counting of individual neuronal cells. Given the small subgroup size, this analysis is exploratory and is likely underpowered to detect moderate between-group differences; results should be interpreted with corresponding caution. Mean HuC/D-positive cells per ganglion were comparable across groups (controls: 11.3 ± 1.6, ODS: 9.3 ± 1.3; STC: 10.0 ± 2.2; ODS + STC: 11.1 ± 1.8; *p* = 0.18), confirming preserved neuronal content within the individual ganglia.

In contrast, linear neuronal density (cells/mm) differed significantly overall (*p* = 0.047) with higher values in the constipation groups (ODS: 21.5 ± 5.2; STC: 20.0 ± 4.6; ODS + STC: 18.5 ± 2.3) compared to controls (13.5 ± 3.5), although pairwise comparisons did not reach significance after correction (*p* = 0.063; Supplementary Fig. 5).

This pattern parallels the increased ganglionic density observed in patients with constipation, particularly in ODS. Neurons per ganglion reflect the neuronal content of individual ganglionic structures, whereas linear neuronal density (cells/mm) additionally incorporates the number of ganglia along the section and is therefore driven jointly by neuronal content and ganglion frequency; the latter, but not the former, differed between groups, indicating that the increase in linear neuronal density reflects a higher number of ganglia rather than neuronal hyperplasia within ganglia (see Supplementary Fig. 5 legend). No correlation was found between MAP2 positivity and HuC/D + cell counts (*ρ* = −0.16, *p* = 0.51), suggesting that these markers capture complementary aspects of neuronal content.

### Immune cell infiltration in the myenteric plexus

**Fig. 4:**
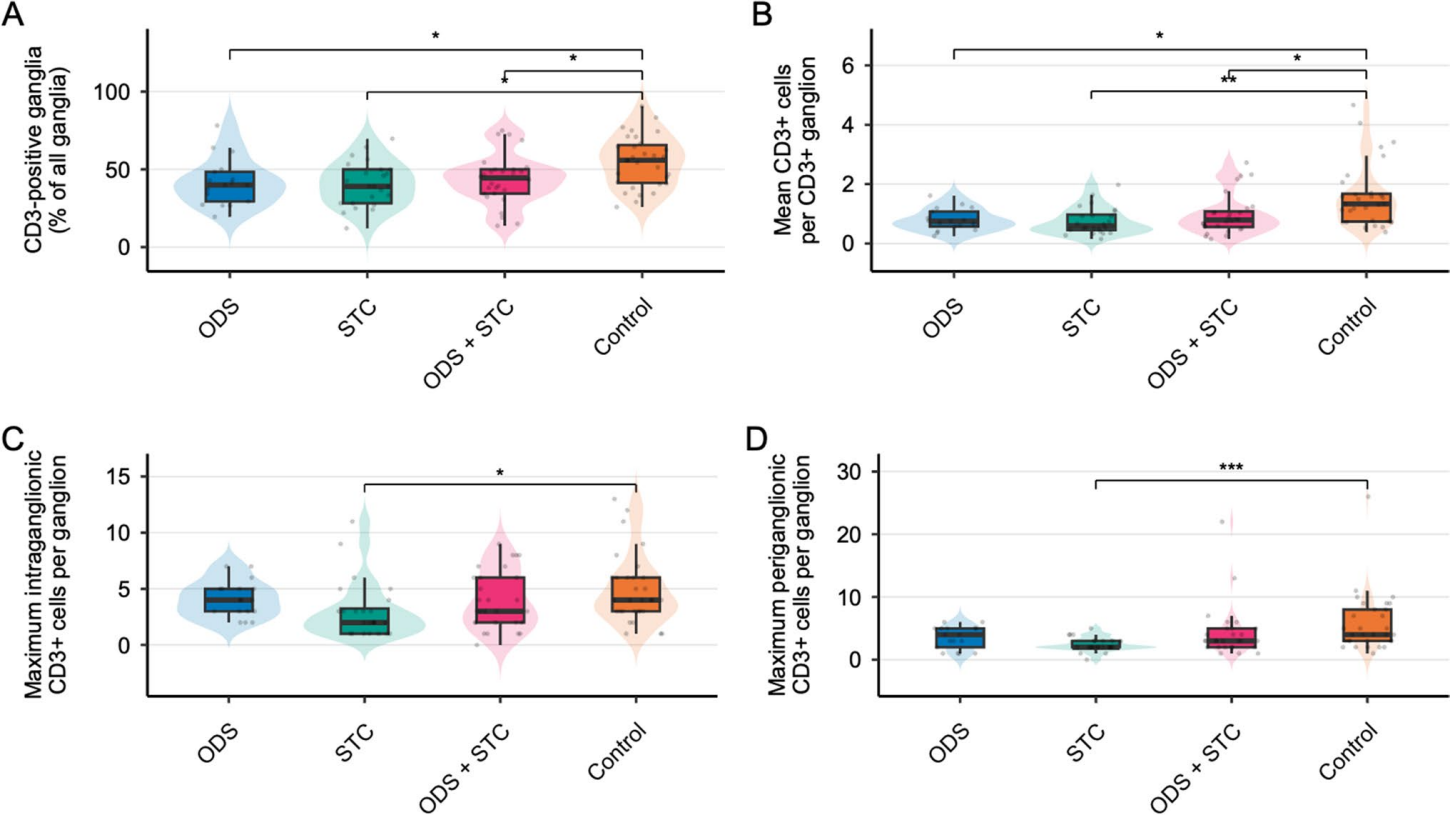
CD3-positive T-lymphocytes in enteric ganglia across patient groups. Comparison of CD3-positive T-lymphocyte distribution in patients with obstructive defecation syndrome (ODS, *n* = 17), slow-transit constipation (STC, *n* = 24), combined ODS + STC (*n* = 28), and controls (*n* = 31). **A** Percentage of ganglia containing CD3-positive T-lymphocytes was reduced in all constipation groups compared to controls (*p < 0.05, **p < 0.01). **B** Mean number of CD3-positive cells per positive ganglion was lower in all constipation groups versus controls (**p < 0.01, ***p < 0.001). **C**, **D** Maximum intra- and periganglionic CD3-positive cell counts were reduced in constipation groups, reaching significance in STC (*p < 0.05, ***p < 0.001). Data are presented as violin plots with superimposed boxplots (median, interquartile range) and individual data points

CD3-positive T-lymphocyte analysis revealed reduced immune cell presence within and around the myenteric plexus in all constipation groups compared to controls (Fig. 4A). The proportion of ganglia containing CD3-positive T-lymphocytes in both constipation groups: ODS (41.8 ± 15.2%; *p* < 0.05), STC (40.5 ± 14.4%; *p* < 0.01), and ODS + STC (43.5 ± 16.2%; *p* < 0.05) was significantly reduced compared to control subjects (55.2 ± 16.6%).

This finding was confirmed by quantitative analysis of the number of CD3-positive T-lymphocytes per positive ganglion (Fig. 4B). Both intraganglionic and periganglionic cell counts were significantly reduced in all constipation groups (ODS: 0.8 ± 0.4, *p* < 0.01; STC: 0.8 ± 0.5, *p* < 0.01; ODS + STC: 1.0 ± 0.7; all *p* < 0.001) compared to controls (1.6 ± 1.1).

Further analysis showed a reduction/loss of the maximum number of CD3-positive T-lymphocytes per ganglion (Fig. 4C) in the constipation groups. The reduction reached significance in intraganglionic counts for STC (3.1 ± 2.6 vs. 4.9 ± 3.1 in controls; *p* < 0.05) and in periganglionic counts (Fig. 4D), which were markedly reduced in STC (2.4 ± 1.1 vs. 5.7 ± 4.7 in controls; *p* < 0.001).

These differences were independent of age and sex (Supplementary Figs. 6 and 7). Overall, functional constipation disorders are associated with reduced presence of CD3-positive T-lymphocytes in the myenteric plexus, most pronounced in STC.

### Exploratory effect size analysis

To complement the *p*-values reported above with a measure of the magnitude of the observed differences, Hedges’ g effect sizes and approximate 95% confidence intervals were calculated post hoc for the principal pairwise comparisons across the morphological, MAP2, HuC/D, and CD3 analyses (Supplementary Table 2). The increased ganglionic density in ODS versus controls corresponded to a large effect (*g* = 1.06 [95% CI 0.44 to 1.69]), whereas the ODS + STC versus control comparison showed a smaller and less precise effect (*g* = 0.35 [95% CI −0.16 to 0.87]), and the STC versus control comparison showed essentially no effect (*g* = −0.02 [95% CI −0.55 to 0.51]), consistent with the pattern of the primary univariate results. The exploratory HuC/D-based linear neuronal density comparisons showed large point estimates in all three constipation subgroups versus controls (*g* = 1.4–1.6), albeit with wide confidence intervals reflecting the small subgroup size (*n* = 5/group). The reduction in CD3-positive T-lymphocyte measures in constipation groups versus controls corresponded to moderate-to-large effects across outcomes (*g* = −0.64 to −0.92), supporting the consistency of this finding across the different CD3-related parameters. As this analysis is based on reported summary statistics rather than raw data, these effect sizes should be interpreted as an exploratory, descriptive complement to the primary analyses.

### Control subgroup homogeneity analysis

Because the surgical control cohort comprised three underlying pathologies (endometriosis, *n* = 9; diverticular disease, *n* = 12; carcinoma, *n* = 10), we performed a post hoc comparison of demographic characteristics and QuPath-derived histomorphological measurements across these subgroups. Age (*p* < 0.001) and ASA classification (*p* < 0.001) differed significantly between subgroups, reflecting their differing clinical profiles, whereas sex (*p* = 0.3) and BMI (*p* = 0.10) did not. In contrast, none of the 12 QuPath-derived measurements of ganglion morphology, MAP2 positivity, or CD3-positive T-lymphocyte infiltration differed significantly between the three control subgroups after correction for multiple testing (Kruskal-Wallis test, Benjamini-Hochberg-adjusted *q* ≥ 0.3 for all comparisons; Supplementary Tables 3 and 4). This suggests that this demographic and clinical heterogeneity did not translate into detectable differences in the myenteric plexus phenotype used as the comparator reference, although the subgroup sizes (*n* = 9–12) limit the power to exclude smaller differences.

## Discussion

This study presents a systematic, standardized, semiautomated digital pathology approach for the quantitative assessment of the myenteric plexus in patients with functional defecation disorders. By establishing standardized methods for clinically well-defined subgroups and comparing these findings with a surgical control cohort, we identified two distinct neuroarchitectural and neuroimmune patterns: increased ganglionic density in patients with obstructive defecation syndrome (ODS) and reduced CD3-positive T-lymphocyte infiltration across all constipation subgroups, with the latter most pronounced in slow-transit constipation.

### Neuroarchitectural differences between ODS and STC

The principal morphological finding was a significantly increased ganglionic density in patients with obstructed defecation syndrome (ODS), whereas patients with slow-transit constipation (STC) had values comparable to those of controls. This observation contrasts with previous studies, which predominantly reported hypoganglionosis and neuronal loss in constipation disorders [13, 14, 23]. Our data, therefore, suggest that chronic constipation cannot be uniformly interpreted as a disorder of neuronal depletion.

Importantly, the increased ganglionic density in ODS did not reflect increased neuronal content within individual ganglia. Both MAP2 immunoreactivity and HuC/D-based neuronal counts demonstrated preserved neuronal content across all groups. The observed increase in linear neuronal density thus resulted from a higher number of ganglia per unit length rather than neuronal hyperplasia within ganglia.

The biological significance of this finding remains uncertain. Potential explanations include developmental variation, adaptive remodeling in response to chronic outlet obstruction, or secondary structural reorganization of the enteric nervous system. However, no conclusions regarding neuronal function or motility pattern can be drawn from the present morphological data alone [13, 24].

### Neuroimmune alterations and CD3-positive T-lymphocytes

The second major finding was a consistent reduction of CD3-positive T-lymphocyte infiltration in the myenteric plexus across all constipation groups, most pronounced in STC, compared to controls. This reduction affected both intra- and periganglionic compartments and was independent of age and sex.

Interpretation of this finding requires careful consideration. In our previous work, we demonstrated CD8-positive T-cell-associated injury of myenteric ganglia, suggesting a potential autoimmune mechanism in the affected patients [19]. One possible, but speculative, explanation for the current observation of reduced CD3-positive T-lymphocytes could be a “burnt-out” inflammatory phase in the later stage of this process. However, this hypothesis is not supported by the neuronal preservation noted above [2].

Alternative explanations for the reduced CD3-positive signal—altered neuroimmune regulation, reduced local recruitment, or secondary remodeling—cannot be excluded, and none, including the burnt-out hypothesis above, can be prioritized from the present cross-sectional data.

Notably, the differences between ODS and STC suggest distinct pathophysiological patterns. While ODS was characterized by increased ganglionic density with relatively little immune involvement compared to controls, STC showed normal ganglionic density but a marked reduction in CD3-positive T-lymphocytes. This divergence supports the concept that ODS and STC, while frequently coexisting, represent distinct pathophysiological entities rather than variations of a single disease process.

### Methodological considerations and relevance

In contrast to prior semi-quantitative approaches, our study applies standardized digital morphometry in line with current recommendations [20, 24, 25]. Thus, this semiautomated digital pathology workflow is a major strength in the present study. Digital ganglion annotation enabled reproducible pixel-based quantification of ganglion density, automated cell detection of neuronal markers, and immune cell distribution. Thus, it reduces inter-observer variability and enables reproducible measurements. The use of multiple complementary markers (EvG for morphology, MAP2 for neuronal cytoplasm, HuC/D for neuronal nuclei, and CD3 for T-lymphocytes) provides a multidimensional characterization of the enteric nervous system that goes beyond descriptive histopathology.

The inclusion of a surgical control cohort allows for contextual interpretation of findings, although interindividual variability within these groups highlights the challenge of defining “normal” reference values in human intestinal tissue.

### Clinical implications

These findings may have clinical implications. Current management of chronic constipation relies primarily on functional testing and symptom-based classification and treatment without incorporating potential structural assessment of the enteric nervous system. If confirmed in larger studies, quantitative assessment of the myenteric plexus could complement the existing diagnostic algorithms and contribute to a more precise phenotypic classification and, potentially, more targeted therapeutic strategies.

At present, however, the functional relevance of these findings remains unclear. Morphological alterations cannot be directly translated into motility dysfunction, and the relationship between structure and function in the enteric nervous system requires further investigation [2].

The role of full-thickness biopsy in this context also warrants caution: current evidence is insufficient to support its routine integration into diagnostic algorithms or surgical decision-making.

Clarifying whether the observed alterations represent primary disease mechanisms or secondary adaptations will require integrative studies (see “Conclusion”). Until then, morphological assessment should be regarded as a promising yet exploratory tool for evaluating functional bowel motility disorders.

### Limitations

Several limitations warrant consideration. First, and most importantly, the control cohort comprised patients undergoing colorectal resection for colorectal cancer, diverticular disease, or endometriosis, rather than healthy individuals. Full-thickness colonic tissue cannot be obtained from truly healthy individuals for ethical reasons, and this constraint applies to essentially all histopathological studies of the human enteric nervous system. Because each of these underlying conditions may itself influence enteric neuronal architecture and the local immune microenvironment, our findings should be interpreted as relative differences between surgical cohorts rather than as deviations from a physiologically normal reference, and not as changes isolated from alterations within the comparator cohort itself; the term “control” is used in this restricted sense throughout the manuscript.

Second, as in most single block sampling strategies, the analysis was based on a single representative tissue block per case and may not capture the variability of myenteric plexus changes along the resected segment. Block selection was based on tissue quality and plexus visibility rather than formal blinding to clinical diagnosis (see “Methods”), which represents an additional potential source of selection bias. Furthermore, linear ganglion density (ganglia/cm), used as the principal density metric, can be influenced by factors unrelated to the true biological density of the enteric nervous system, including the plane of section and tissue orientation, bowel wall thickness, tissue shrinkage, and variability in section geometry; a formal stereological approach was not undertaken. While this metric was applied consistently across all groups, these methodological factors should be considered when interpreting the magnitude, though not necessarily the direction, of the observed differences, which represents the principal finding underlying our conclusions.

Third, interstitial cells of Cajal were not assessed, although they play a critical role as pacemakers in intestinal motility and have been implicated in postoperative outcomes following STARR procedures [19]. Fourth, histomorphological findings were not correlated with functional motility data, limiting the interpretation of the physiological significance of the observed changes. Fifth, the HuC/D-based neuronal quantification was performed in only five patients per group; this exploratory analysis is likely underpowered, and its findings should not be over-interpreted.

Sixth, statistical comparisons were primarily univariate; given the heterogeneous composition of the surgical cohorts, we cannot fully exclude residual confounding by age, sex, type of surgery, or underlying control pathology; a formal multivariable adjustment for these factors is not feasible from the available data due to size and heterogeneity of the subgroups. A direct post hoc comparison, however, showed that although the three control subgroups differed significantly in age and ASA classification, none of the QuPath-derived histomorphological measurements differed significantly between them after correction for multiple testing (see “Results,” “Control subgroup homogeneity analysis”; Supplementary Tables 3 and 4), suggesting that this heterogeneity did not materially bias the histomorphological reference values used for the principal group comparisons, although residual or unmeasured confounding cannot be fully excluded. Finally, the single-center design may limit generalizability.

## Conclusion

This study establishes a standardized digital approach for quantitative assessment of the myenteric plexus and suggests distinct neuroarchitectural and neuroimmune patterns in functional defecation disorders, derived from comparison with a surgical rather than a healthy control cohort. The identification of distinct morphological phenotypes in ODS versus STC may have potential clinical implications. Increased ganglionic density in ODS and reduced presence of CD3-positive T-lymphocytes, particularly in STC, may indicate distinct underlying pathophysiological processes in these clinically overlapping conditions, although these associations require external validation in independent cohorts before mechanistic or causal conclusions can be drawn. These findings provide a basis for future studies integrating morphological, functional, and molecular analyses to improve understanding and management of chronic constipation.

## Supplementary Information

Below is the link to the electronic supplementary material.

ESM 1 PDF (862 KB)

## Data Availability

The anonymized data supporting the findings of this study are available from the corresponding author upon reasonable request.

## Declarations

### Declaration of AI and AI-assisted assisted technologies in the writing process

During the preparation of this work, the authors used ChatGPT (OpenAI) to improve the readability and language of the manuscript (AI-assisted copy editing). After using this tool, the authors reviewed and edited the content as needed and take full responsibility for the content of the publication. All authors read and approved the final manuscript and agree to be accountable for all aspects of the work in ensuring that questions related to the accuracy or integrity of any part of the work are appropriately investigated and resolved. The final version reflects their original work.

### Submission declaration and verification

This manuscript has not been published previously and is not under consideration for publication elsewhere. The manuscript is approved by all authors and by the responsible authorities where the work was carried out. If our work will be accepted, it will not be published elsewhere in the same form, in English or in any other language, including electronically, without the written consent of the copyright holder.
