# Supplementary material for: Distinct enteric nervous system pattern in obstructed defecation syndrome and slow-transit constipation: a controlled digital pathology study
Source: Int J Colorectal Dis. 2026 Aug 3;41(1):127. doi: 10.1007/s00384-026-05209-4 (PMC13433575; doi:10.1007/s00384-026-05209-4)
Supplement: Supplementary file 1 [file 384_2026_5209_MOESM1_ESM.pdf]

Supplementary material

Supplementary tables

Supplementary Table 1. Quantitative analysis of myenteric plexus characteristics

Morphometric and immunohistochemical parameters in patients with obstructive defecation syndrome (ODS, n=17), slow transit constipation (STC, n=24), combined ODS+STC (n=28), and controls (n=31). Data are presented as mean ± SD. Significant differences ($p<0.05$) are highlighted. Parameters include ganglionic morphology, MAP2-based neuronal content, and CD3+ lymphocytes infiltration. Corresponding data are visualized in Figures 2-4

Measurement	Overall (n=100)	ODS (n=17)	STC (n=24)	ODS + STC (n=28)	Controls (n=31)	p-value
**Mean ganglia/cm**	20.0 (5.3)	23.8 (5.0)	18.5 (4.4)	20.5 (5.9)	18.6 (4.7)	**0.002***
**Mean ganglion area (μm^2)**	16,313.9 (5,919.2)	15,015.2 (5,138.2)	15,424.0 (5,491.8)	17,871.6 (5,394.5)	16,308.3 (6,955.3)	0.3
**Mean ganglion circularity**	0.6 (0.1)	0.6 (0.0)	0.6 (0.1)	0.6 (0.1)	0.5 (0.1)	**0.045***
**Maximum ganglion diameter (μm)**	940.3 (369.5)	813.1 (306.2)	939.3 (323.7)	940.4 (264.8)	1,010.7 (492.7)	0.4
**Mean MAP2 positivity (% of all ganglia)**	13.4 (5.1)	12.4 (4.6)	14.4 (6.1)	13.1 (5.2)	13.5 (4.6)	0.6
**MAP2-positive ganglia (% of all ganglia)**	89.5 (8.2)	87.8 (6.8)	90.9 (9.5)	90.7 (7.6)	88.4 (8.4)	0.2
**CD3-positive ganglia (% of all ganglia)**	46.1 (16.7)	41.8 (15.2)	40.5 (14.4)	43.5 (16.2)	55.2 (16.6)	**0.006***
**Mean CD3+ cells per CD3+ ganglion (total)**	1.1 (0.8)	0.8 (0.4)	0.8 (0.5)	1.0 (0.7)	1.6 (1.1)	**<0.001***
**Mean CD3+ cells per CD3+ ganglion (ganglionic)**	0.5 (0.3)	0.4 (0.2)	0.4 (0.3)	0.5 (0.3)	0.6 (0.4)	0.068
**Mean CD3+ cells per CD3+ ganglion (periganglionic)**	0.6 (0.6)	0.4 (0.2)	0.4 (0.2)	0.5 (0.4)	1.0 (0.8)	**<0.001***
**Maximum intraganglionic CD3+ cells per ganglion**	4.1 (2.7)	4.1 (1.6)	3.1 (2.6)	4.0 (2.6)	4.9 (3.1)	**0.032***
**Maximum periganglionic CD3+ cells per ganglion**	4.2 (3.8)	3.5 (1.8)	2.4 (1.1)	4.3 (4.2)	5.7 (4.7)	**0.001***

Supplementary Table 2. Exploratory effect sizes for principal pairwise group comparisons

This table presents an exploratory, post hoc supplementary analysis. Hedges' g effect sizes and approximate 95% confidence intervals were calculated from the group means, standard deviations, and sample sizes reported in the Results, using pooled-SD Cohen's d with small-sample (Hedges) correction. Because the original group comparisons were performed with non-parametric tests (Kruskal-Wallis / Wilcoxon), these values are approximations intended to convey the magnitude of observed differences and should be interpreted as complementary to, not a replacement for, the reported p-values.

Comparison	Outcome	Mean difference [95% CI]	Hedges' g [95% CI]
**ODS vs Control**	Ganglion density (ganglia/cm)	5.20 [2.30 to 8.10]	1.06 [0.44 to 1.69]
**ODS+STC vs Control**	Ganglion density (ganglia/cm)	1.90 [-0.84 to 4.64]	0.35 [-0.16 to 0.87]
**STC vs Control**	Ganglion density (ganglia/cm)	-0.10 [-2.52 to 2.32]	-0.02 [-0.55 to 0.51]
**ODS vs STC**	Ganglion density (ganglia/cm)	5.30 [2.34 to 8.26]	1.12 [0.45 to 1.78]
**ODS vs Control**	HuC/D neurons/ganglion (exploratory, n=5/grp)	-2.00 [-3.81 to -0.19]	-1.24 [-2.59 to 0.11]
**STC vs Control**	HuC/D neurons/ganglion (exploratory, n=5/grp)	-1.30 [-3.68 to 1.08]	-0.61 [-1.88 to 0.66]
**ODS+STC vs Control**	HuC/D neurons/ganglion (exploratory, n=5/grp)	-0.20 [-2.31 to 1.91]	-0.11 [-1.35 to 1.13]
**ODS vs Control**	HuC/D linear density (cells/mm, exploratory)	8.00 [2.51 to 13.49]	1.63 [0.20 to 3.06]
**STC vs Control**	HuC/D linear density (cells/mm, exploratory)	6.50 [1.43 to 11.57]	1.44 [0.05 to 2.83]
**ODS+STC vs Control**	HuC/D linear density (cells/mm, exploratory)	5.00 [1.33 to 8.67]	1.52 [0.12 to 2.93]
**ODS vs Control**	% ganglia with CD3+ lymphocytes	-13.40 [-22.69 to -4.11]	-0.82 [-1.43 to -0.20]
**STC vs Control**	% ganglia with CD3+ lymphocytes	-14.70 [-22.91 to -6.49]	-0.92 [-1.48 to -0.36]
**ODS+STC vs Control**	% ganglia with CD3+ lymphocytes	-11.70 [-20.08 to -3.32]	-0.70 [-1.23 to -0.18]
**ODS vs Control**	CD3+ cells/positive ganglion	-0.80 [-1.23 to -0.37]	-0.86 [-1.47 to -0.24]
**STC vs Control**	CD3+ cells/positive ganglion	-0.80 [-1.24 to -0.36]	-0.89 [-1.44 to -0.33]
**ODS+STC vs Control**	CD3+ cells/positive ganglion	-0.60 [-1.07 to -0.13]	-0.64 [-1.16 to -0.11]
**STC vs Control**	Max intraganglionic CD3+ count	-1.80 [-3.31 to -0.29]	-0.61 [-1.16 to -0.07]
**STC vs Control**	Max periganglionic CD3+ count	-3.30 [-5.01 to -1.59]	-0.90 [-1.46 to -0.34]

Supplementary Table 3 Demographic characteristics of the control cohort, stratified by underlying pathology

Demographic characteristics of the 31 surgical control patients, stratified by underlying pathology (endometriosis, diverticular disease, carcinoma). p-values: Kruskal-Wallis rank sum test (continuous variables) or Fisher's exact test (categorical variables).

Characteristic	Overall (n=31)	Endometriosis (n=9)	Diverticular disease (n=12)	Carcinoma (n=10)	p-value
**Age at surgery (years), mean (SD)**	57.6 (21.5)	31.9 (8.5)	58.4 (12.9)	79.8 (7.9)	<0.001
**Sex, female, n (%)**	25 (80.6%)	9 (100.0%)	9 (75.0%)	7 (70.0%)	0.3
**BMI (kg/m²), mean (SD)**	23.8 (4.7)	21.7 (1.4)	25.8 (6.3)	23.3 (3.6)	0.10
**ASA classification ≥ III, n (%)**	13 (41.9%)	1 (11.1%)	2 (16.7%)	10 (100.0%)	<0.001

Supplementary Table 4 QuPath-derived histomorphological measurements of the control cohort, stratified by underlying pathology

Quantitative myenteric plexus measurements (as in Supplementary Table 1) among the 31 surgical control patients, stratified by underlying pathology. Data are presented as mean (SD). p-values: Kruskal-Wallis rank sum test; q-values: Benjamini-Hochberg-adjusted p-values across the twelve measurements. None of the measurements differed significantly between subgroups after correction for multiple testing.

Measurement	Overall (n=31)	Endometriosis (n=9)	Diverticular disease (n=12)	Carcinoma (n=10)	p-value	q-value
**Mean ganglia/cm**	18.6 (4.7)	20.1 (5.5)	18.4 (4.2)	17.7 (4.6)	0.6	0.9
**Mean ganglion area (µm²)**	16,308.3 (6,955.3)	17,616.4 (8,773.4)	17,998.7 (5,204.1)	13,102.4 (6,541.5)	0.066	0.3
**Mean ganglion circularity**	0.5 (0.1)	0.5 (0.1)	0.6 (0.1)	0.5 (0.1)	0.5	0.9
**Maximum ganglion diameter (µm)**	1,010.7 (492.7)	1,104.7 (525.2)	1,172.0 (508.4)	732.4 (344.5)	0.067	0.3
**Mean MAP2 positivity (% of ganglion area)**	13.5 (4.6)	11.0 (3.5)	13.7 (3.0)	15.4 (6.3)	0.10	0.3
**MAP2-positive ganglia (% of all ganglia)**	88.4 (8.4)	83.3 (10.8)	88.8 (5.9)	92.5 (6.6)	0.048	0.3
**CD3-positive ganglia (% of all ganglia)**	55.2 (16.6)	55.0 (15.7)	58.8 (18.8)	51.0 (15.0)	0.6	0.9
**Mean CD3+ cells per CD3+ ganglion (total)**	1.6 (1.1)	1.5 (1.0)	1.6 (1.2)	1.5 (1.0)	>0.9	>0.9
**Mean CD3+ cells per CD3+ ganglion (ganglionic)**	0.6 (0.4)	0.5 (0.3)	0.7 (0.4)	0.6 (0.5)	0.7	0.9
**Mean CD3+ cells per CD3+ ganglion (periganglionic)**	1.0 (0.8)	1.0 (0.9)	1.0 (0.9)	0.9 (0.6)	>0.9	>0.9
**Maximum intraganglionic CD3+ cells per ganglion**	4.9 (3.1)	5.4 (3.9)	4.8 (2.0)	4.6 (3.5)	0.8	>0.9
**Maximum periganglionic CD3+ cells per ganglion**	5.7 (4.7)	5.7 (3.1)	5.7 (6.7)	5.9 (3.5)	0.6	0.9

Supplementary figures

Supplementary Fig 1 Enteric ganglion characteristics stratified by age

Comparison of enteric ganglion parameters in obstructive defecation syndrome (ODS, n=17), slow transit constipation (STC, n=24), combined ODS+STC (n=28), and controls (n=31), stratified by age (<45, 45-65, and >65 years). **a:** Ganglionic density differed between patient groups (p=0.00145) but not by age. **b and d:** Mean ganglion area and maximum diameter varied by age (p=0.0441 and p=0.004) but not by patient group. **c:** Mean ganglion circularity showed differences between patient groups (p=0.0294) but not by age. Data are represented as boxplots (median, interquartile range) with individual data points

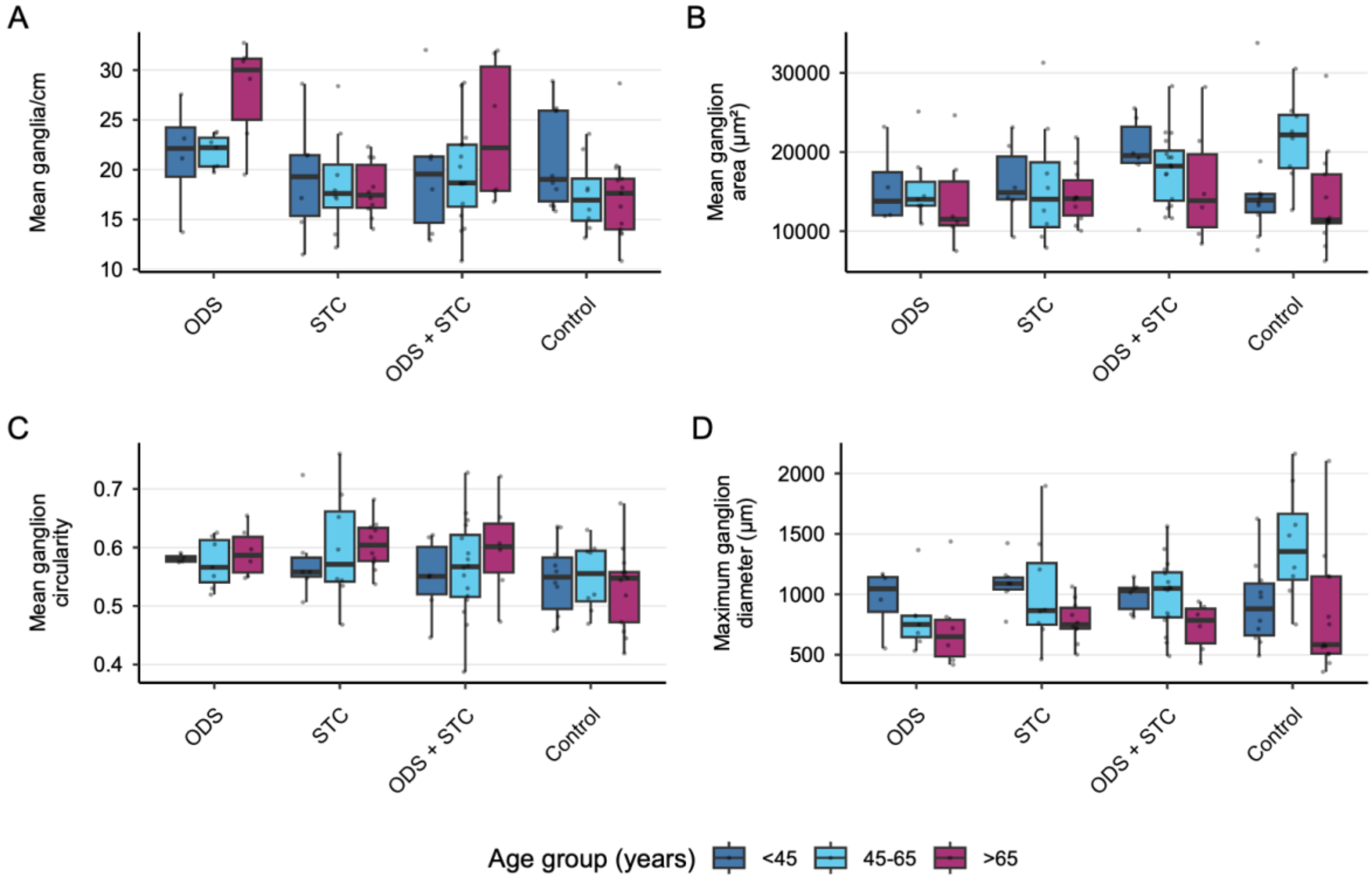

Supplementary Fig 2 Enteric ganglion characteristics stratified by sex

Comparison of enteric ganglion parameters in obstructive defecation syndrome (ODS, n=17), slow transit constipation (STC, n=24), combined ODS+STC (n=28), and controls (n=31), stratified by sex (male, female).
a: Ganglionic density differed between patient groups (p=0.004) but not by sexes, with no significant interaction. **b and d:** Mean ganglion area and maximum diameter showed no significant differences by group or sex. **c:** Mean ganglion circularity differed between patient groups (p=0.018) but not by sex. Data are presented as boxplots (median, interquartile range) and individual data points

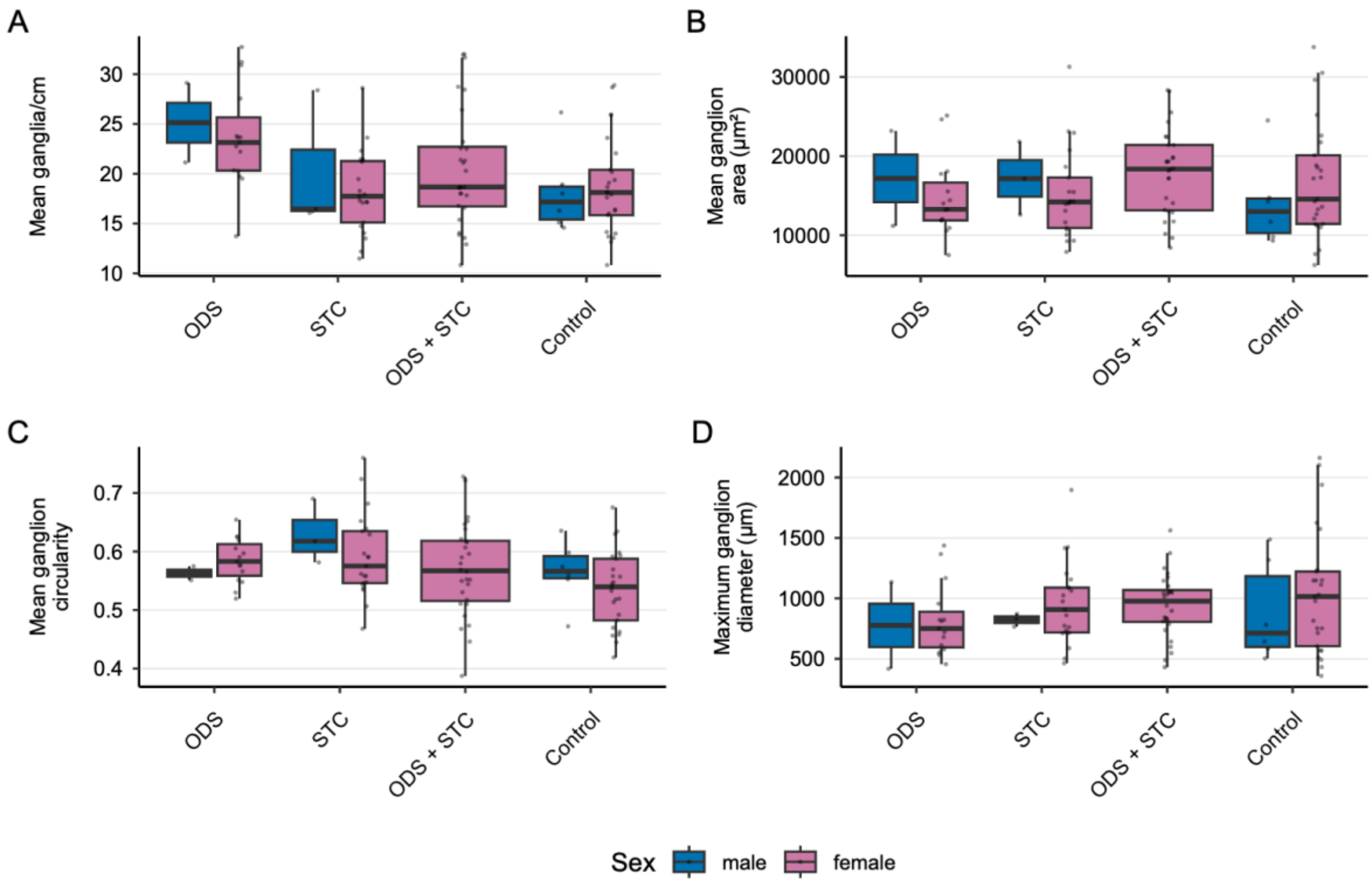

Supplementary Fig 3 MAP2 positivity stratified by age
Comparison of MAP2 immunoreactivity in obstructive defecation syndrome (ODS, n=17), slow transit constipation (STC, n=24), combined ODS+STC (n=28), and controls (n=31), stratified by age (<45, 45-65, and >65 years). a**:** Mean MAP2 positivity showed no differences between patient groups or age categories, with no significant interaction effect. **b:** Percentage of MAP2-positive ganglia showed a non-significant trend across age groups with no interaction effects. Data are presented as boxplots (median, interquartile range) and individual data points

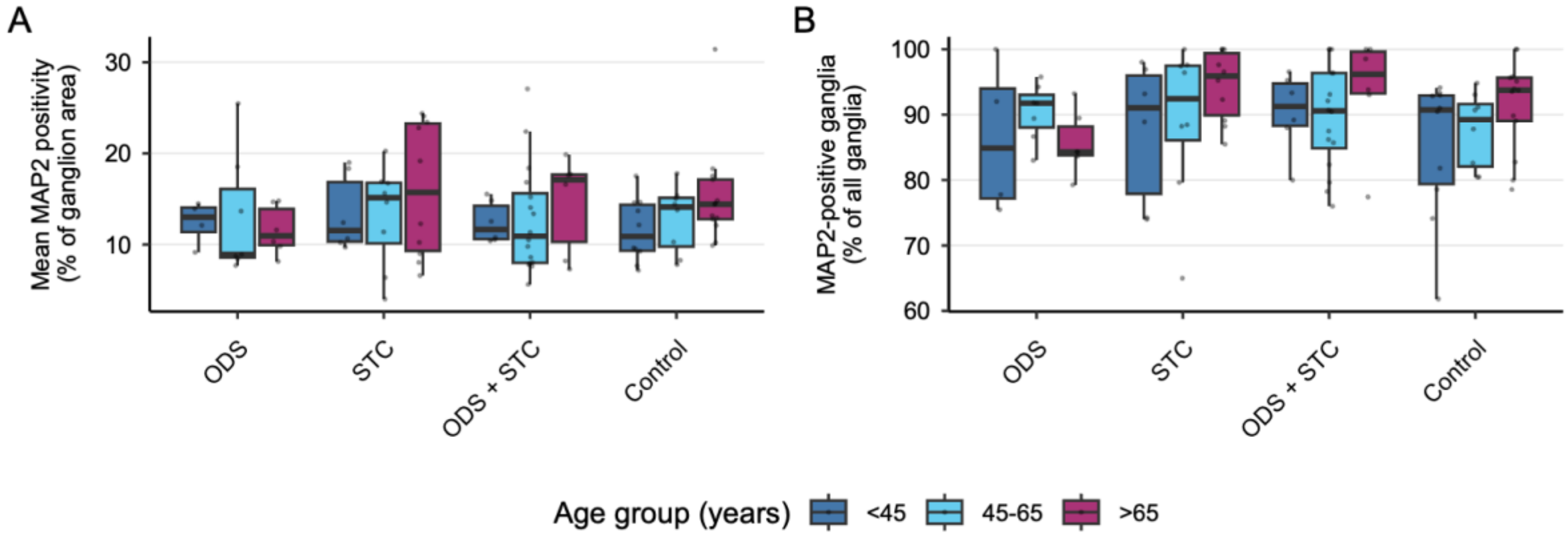

Supplementary Fig 4 MAP2 positivity stratified by sex
Comparison of MAP2 immunoreactivity in obstructive defecation syndrome (ODS, n=17), slow transit constipation (STC, n=24), combined ODS + STC (n=28), and controls (n=31), stratified by sex (male, female). **a:** Mean MAP2 positivity showed no differences between patient groups or sex, with no significant interaction effect. **b:** Percentage of MAP2-positive ganglia showed no group or sex differences, with a non-significant interaction. Data are presented as boxplots (median, interquartile range) and individual data points

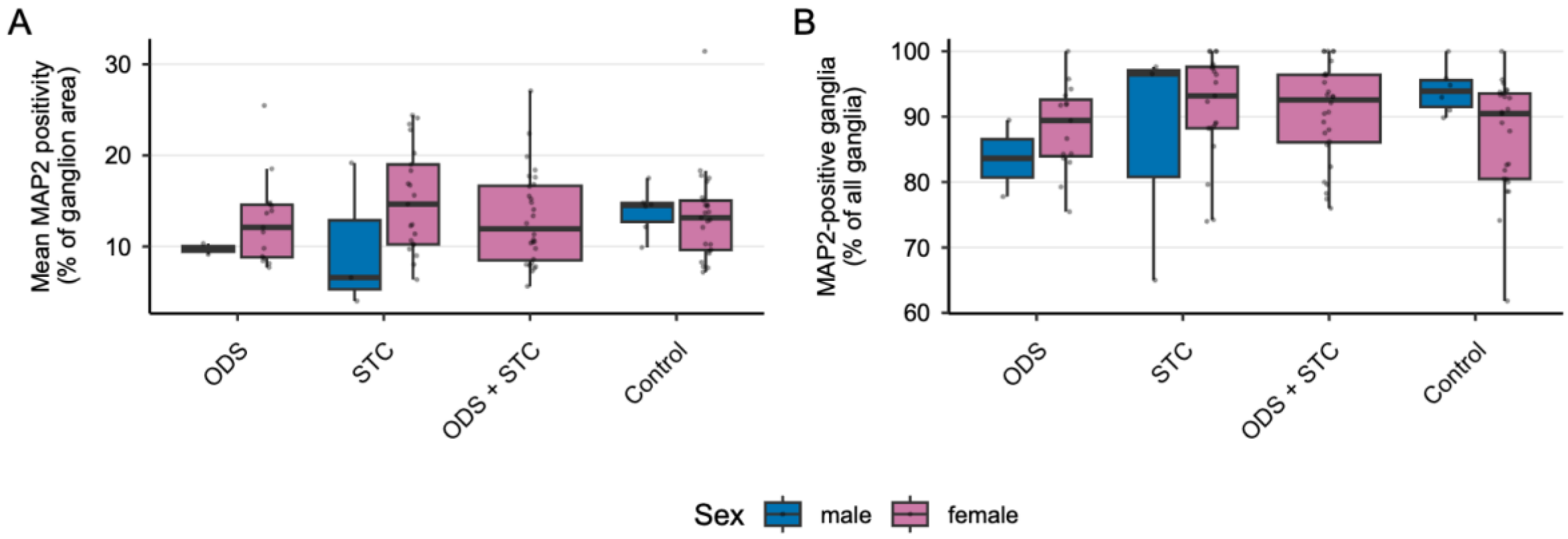

Supplementary Fig 5 Comparison of HuC/D immunoreactivity in a subset of patients
Comparison of neuronal nuclear marker HuC/D in ODS, STC, combined ODS+STC, and controls (5 per group) **a:** Mean HuC/D-positive cells per ganglion did not differ between groups p=0.18). **b:** Linear neuronal density (HuC/D-positive cells/mm) differed between groups (p=0.047), with elevated values in all constipation groups, although pairwise comparisons reached no significance after correction. Data are presented as violin plots with superimposed boxplots (median, interquartile range) and individual data points

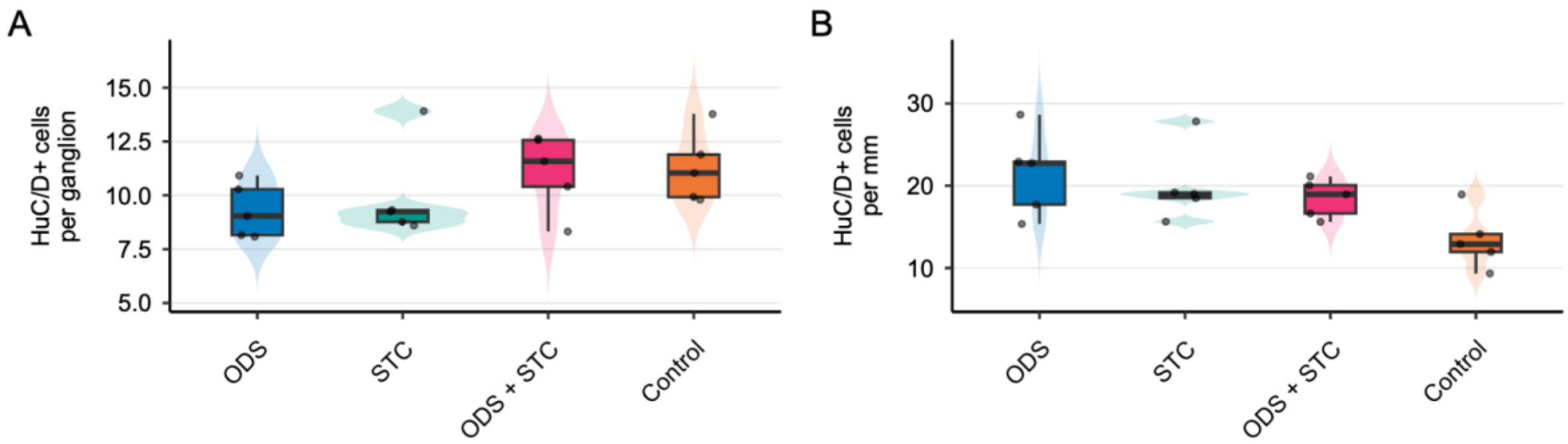

Supplementary Fig 6 CD3-positive lymphocytes stratified by age
Comparison of CD3+ T lymphocyte distribution in obstructive defecation syndrome (ODS, n=17), slow transit constipation (STC, n=24), combined ODS+STC (n=28), and controls (n=31), stratified by age (<45, 45-65, and >65 years). **a and b:** Percentage of CD3+ ganglia and mean CD3+ cells per ganglion differed between patient groups (p=0.004) but not by age, with no interaction effect. **c:** Maximum number of intraganglionic CD3+ cells showed no significant differences. **d:** Maximum number of periganglionic CD3+ cells differed between groups (p<0.01) but not by age. Data are presented as boxplots (median, interquartile range) with individual data points

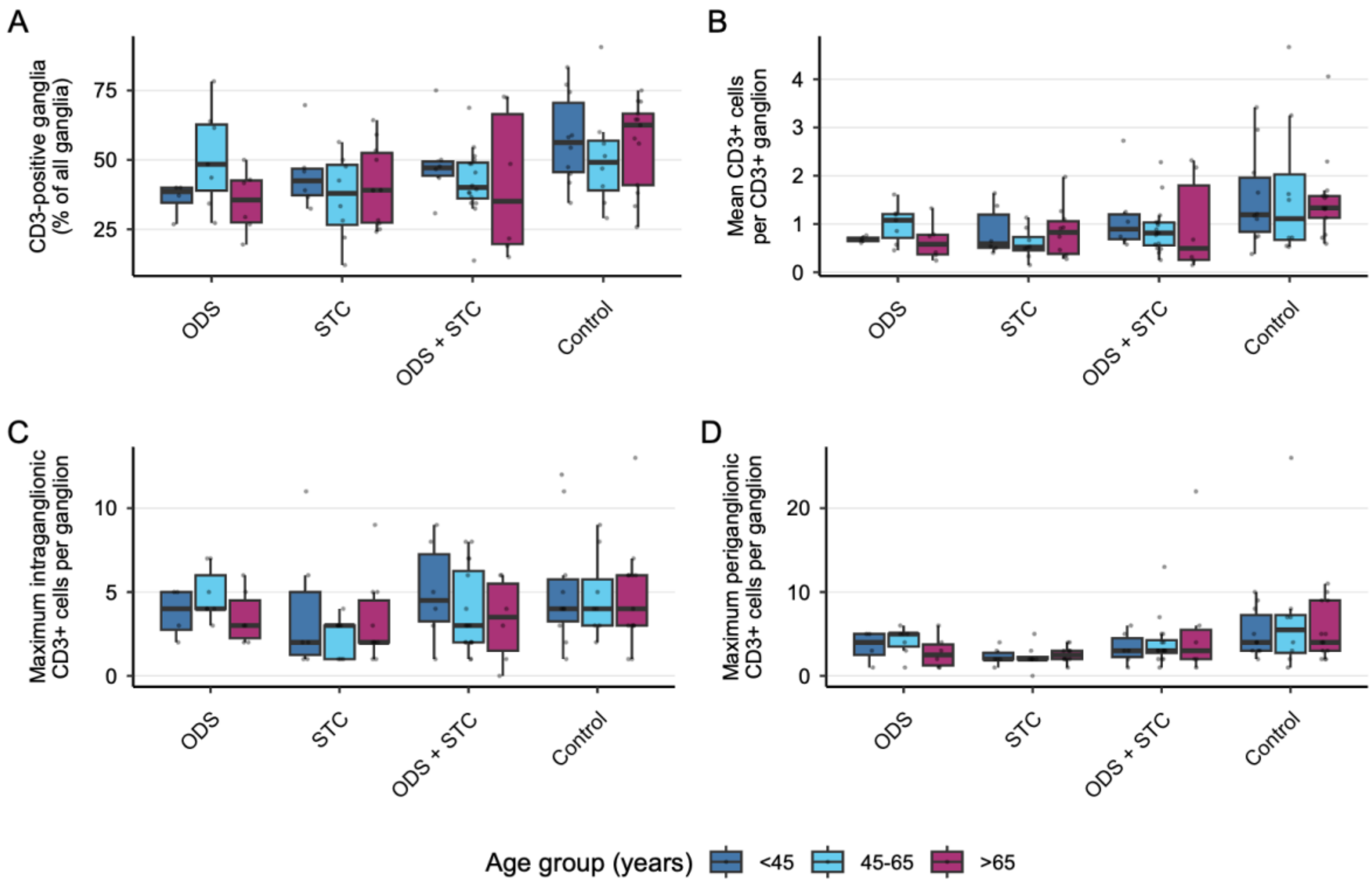

Supplementary Fig 7 CD3-positive lymphocytes stratified by sex
Comparison of CD3+ T lymphocyte distribution in obstructive defecation syndrome (ODS, n=17), slow transit constipation (STC, n=24), combined ODS +STC (n=28), and controls (n=31), stratified by sex (male, female).
A and B: Proportion of CD3+ ganglia and mean CD3+ cells per ganglion differed between patient groups ($p=0.002$ and $p=0.0005$) but not by sex, and with no interaction effect **C:** Maximum number of intraganglionic CD3+ cells showed no significant differences. **D:** Maximum number of periganglionic CD3+ cells differed between groups ($p<0.01$), with a non-significant interaction trend 8pbetween patient group and sex ($p\approx0.09$) and no main effect of sex. Data are presented as boxplots (median, interquartile range) with individual data points

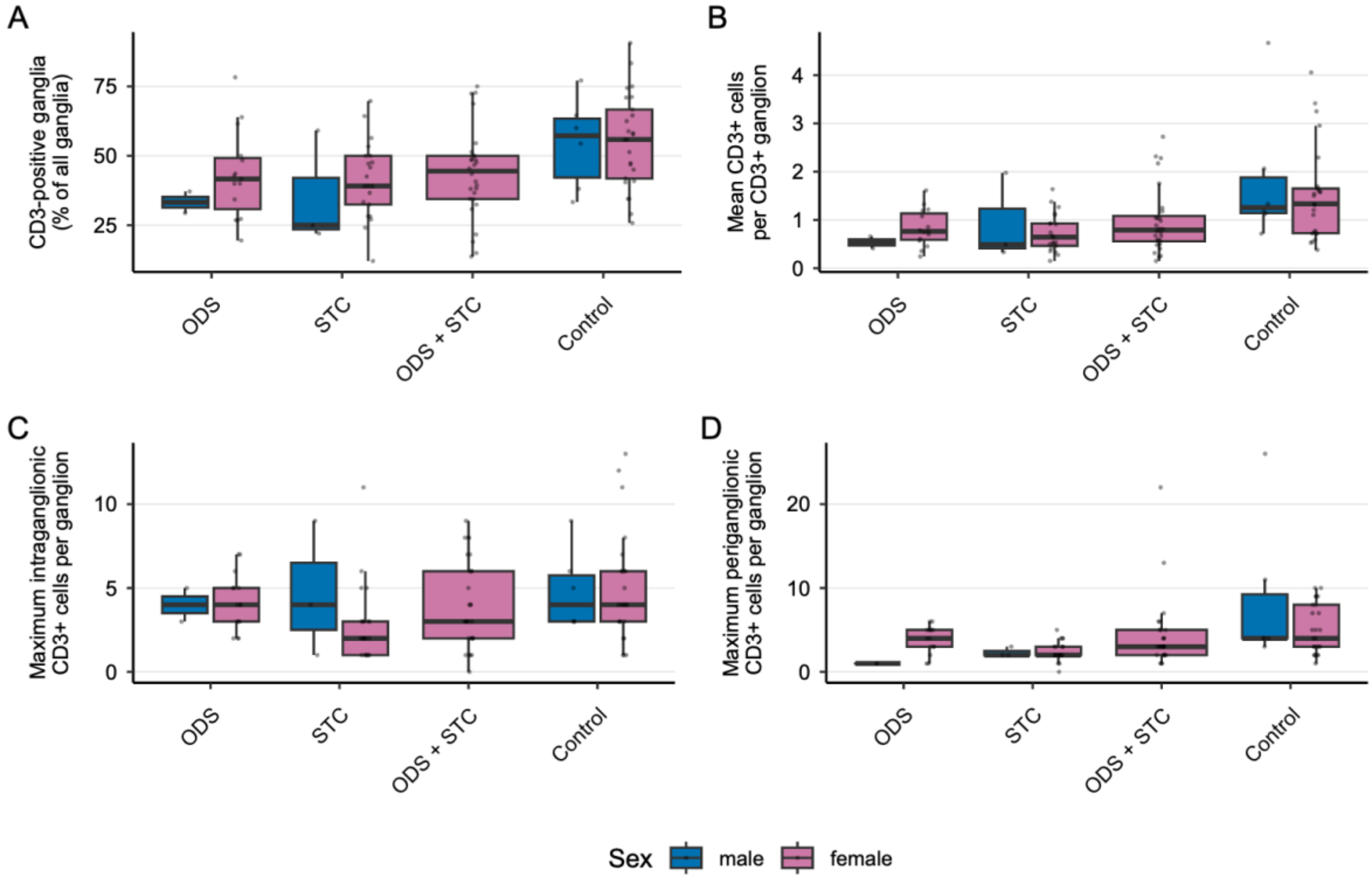
A
CD3-positive ganglia (% of all ganglia)
25
50
75
B
Mean CD3+ cells per CD3+ ganglion
0
1
2
3
4
C
Maximum intraganglionic CD3+ cells per ganglion
0
5
10
D
Maximum periganglionic CD3+ cells per ganglion
0
10
20
ODS
STC
ODS + STC
Control
Sex
male
female
